# Neoadjuvant Chemotherapy Followed by Surgery versus Surgery Alone for Gastric Carcinoma: Systematic Review and Meta-Analysis of Randomized Controlled Trials

**DOI:** 10.1371/journal.pone.0086941

**Published:** 2014-01-30

**Authors:** A-Man Xu, Lei Huang, Wei Liu, Shuang Gao, Wen-Xiu Han, Zhi-Jian Wei

**Affiliations:** 1 Anhui Medical University, Hefei, China; 2 Department of General Surgery, the First Affiliated Hospital of Anhui Medical University, Hefei, China; 3 Guangdong Provincial Key Laboratory of Liver Disease Research, The Third Affiliated Hospital of Sun Yat-sen University, Guangzhou, China; 4 Department of Medical Oncology, the First Affiliated Hospital of Anhui Medical University, Hefei, China; The Norwegian University of Science and Technology (NTNU), Norway

## Abstract

**Background:**

The effect of neoadjuvant chemotherapy (NAC) on Gastric carcinoma (GC) has been extensively studied, while its survival and surgical benefits remain controversial. This study aims to perform a meta-analysis of high-quality randomized controlled trials (RCTs), comparing efficacy, safety and other outcomes of NAC followed by surgery with surgery alone (SA) for GC.

**Methods:**

We systematically searched databases of MEDLINE, EMBASE, The Cochrane Library and Springer for RCTs comparing NAC with SA when treating GC. Reference lists of relevant articles and reviews, conference proceedings and ongoing trial databases were also searched. Primary outcomes were 3-year and 5-year survival rates, survival time, and total and perioperative mortalities. Secondary outcomes included down-staging effects, R0 resection rate, and postoperative complications. Meta-analysis was conducted where possible comparing items using relative risks (RRs) and weighted mean differences (WMDs) according to type of data. NAC-related objective response, safety and toxicity were also specifically analyzed.

**Results:**

A total of 9 RCTs comparing NAC (n = 511) with SA (n = 545) published from 1995 to 2010 were identified. SA tended to be accompanied with higher overall mortality rate than NAC (46.03% vs 40.61%, RR: 0.83, 95% CI: 0.65–1.06, *P* = 0.14). Significantly, higher incidence of cases without regional lymph node metastasis observed upon resection were achieved among patients receiving NAC than those undergoing SA (25.68% vs 16.95%, RR: 1.92, 95% CI: 1.20–3.06, *P* = 0.006). All other parameters were comparable. Of the evaluable patients, 43.0% demonstrated either complete or partial response. The comprehensive NAC-related side-effect rate was 18.2% among patients available for safety assessment.

**Conclusions:**

NAC contributes to lowering nodal stages, and potentially reduces overall mortality. Response rate may be an important influential factor impacting advantages, with chemotherapy-related adverse effects as a drawback. This level 1a evidence doesn't support NAC to outweigh SA in terms of survival and surgical benefits when dealing with GC.

## Introduction

Although the incidence and cancer-related mortality have been decreasing steadily during the past century, gastric carcinoma (GC) remains one of the most common malignancies, and the second leading cause of cancer death worldwide [1]–[3]. Approximately 2/3 of GC patients are at advanced stages when initially diagnosed [4], with a 5-year survival rate of about 25% [5].

For locally advanced lesion, adjuvant chemoradiotherapy is preferred in the US and Canada [6], and the standard treatment is pre- or post-operative chemotherapy in Europe, chemotherapy and D2 gastrectomy in Asia, D2 plus postoperative chemotherapy with S-1 (1 M tegafur−0.4 M gimestat-1 M ostat potassium) for 1 year in Japan, and D2 plus postoperative chemotherapy with capecitabine and oxaliplaitn for around 6 months in Korea [7]–[11]. R0 resection was aimed for by gastrectomy with standard D2 lymphadenectomy [10]. However, even with D2 gastrectomy and adjuvant chemotherapy with S-1, the prognosis of stage 3 tumor is not satisfactory [12].

Adjuvant therapy for GC has been extensively studied, and a recently published meta-analysis demonstrated a small but statistically significant, affirmative and absolute 7% benefit in overall survival for patients treated with 5-fluorouracil (5-FU)-based adjuvant chemotherapy versus SA for locally advanced GC [13].

Neoadjuvant chemotherapy (NAC) has gained increasing attention as a treatment for GC since Wilke [14] first reported its application in the management of GC in 1989. NAC, defined as the chemotherapy supplied before surgery, has been tested in diverse trials, while its role for GC patients remains controversial with conlicting results revealed [13], [15], [16]. However, practice and robustly assess claims of NAC with perioperative and survival benefits are unsubstantiatedly informed with weak and insufficient evidence base. Many other controversies remain, including down-staging effect and presence of tumor-free resection margin (R0 resection), which have kept unsolved largely because most comparisons between NAC and SA for GC had been reported as parts of retrospective and observational studies until these RCTs analyzed in our study emerged.

Up till now, pooled analyses on effectiveness of NAC for only GC patients have been conducted by Liao [17], Ge [18], Li [19] and Wu [20]. However, their studies are accompanied with significant drawbacks. Liao's analysis [17] based on limited evidences included one trial [21] with imbalanced post-surgical chemotherapy, adding obvious bias to the combined results. Ge's study [18] misjudged one nonrandomized observational trial [22] for RCT, and also included 2 researches [8], [23] with mismatched post-operative handling, thus his view that NAC can safely improve overall survival rate and improve rates of R0 resection also raises doubt, which should be interpreted with caution. Li's [19] analysis aiming at revealing NAC's role for gastric suffers also enrolled studies [21], [23], [24] with patients having esophageal and gastroesophageal junction cancer who received uneven postoperative chemotherapy with inseparable data, and were also based on non-RCTs, therefore persuasiveness of his conclusion that NAC can improve tumor stage and survival rate of patients with a rather good safety are greatly weakened, and his results also requires reconsideration. Moreover, none of them conducted satisfactory search for sufficient and eligible literatures. Wu's analysis [20] was carried out in the early time with few available qualified RCTs enrolled.

In our study, potential benefits of two managements were quantified using the meta-analytical method. Meta-analysis reaches the highest level of evidence when pooling data only from randomized trials [25], therefore our study which is carried out according to Preferred Reporting Items for Systematic reviews and Meta-Analysis (PRISMA) [26], [27] guidelines and based on intention-to-treat analysis systematically reviewing all the available high-quality RCTs comparing NAC with SA to perform an updated evaluation creates the highest level of evidence.

## Methods

### Literature Search

**Figure 1:**
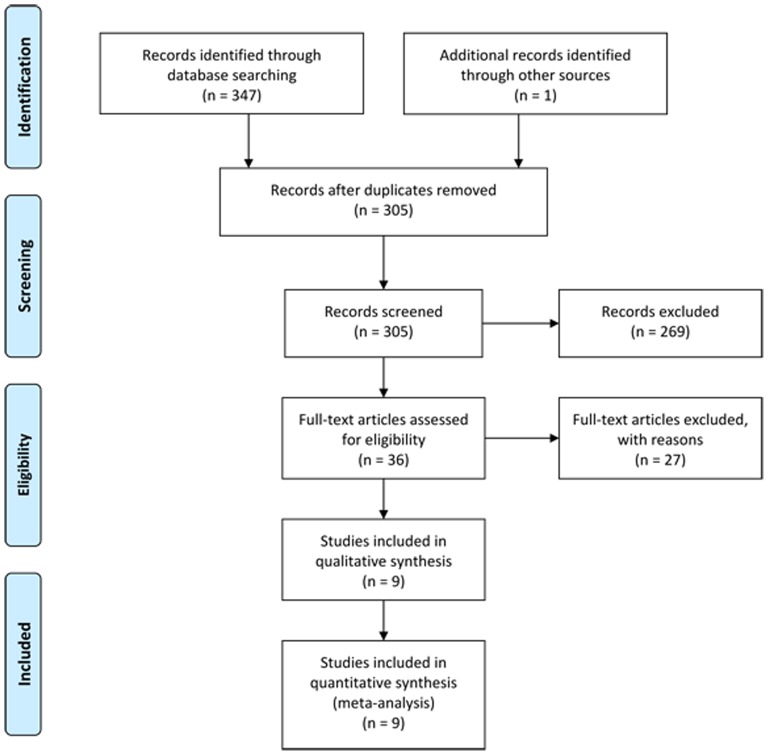
Literature selection flowchart. NAC, neoadjuvant chemotherapy; SA, surgery alone; RCT, randomized controlled trial.

A systematic literature search with search terms “neoadjuvant/preoperative chemotherapy”, “surgery” and “gastric/stomach cancer/carcinoma/adenocarcinoma”, and their combinations as key words was performed in MEDLINE, EMBASE, the Cochrane Library and Springer databases, and Google Scholar (Fig. 1). Special database functions like “related articles” and “explosion” were used to maximize our search and cross-references, references from relevant articles and reviews were also screened. We also searched conference proceedings and ongoing trial databases. Language restrictions were not applied. The last search was performed on July 26^th^, 2013.

### Inclusion Criteria

Titles and abstracts of all identified articles were screened and we selected studies according to the following criteria: population-patients with GC (diagnosed and classified as proposed by Japanese Gastric Cancer Association [28]) without age, gender and racial limitations; intervention and comparative intervention-clearly documented NAC versus SA for GC, regardless of detailed NAC regimen and surgical method applied, grade, classification and position of the lesion; outcomes-at least one of the outcome measures reported below; study design-published and unpublished RCTs.

### Exclusion Criteria

According to the theory of site-dependent differences in tumor biology and genomic [29], we included only GC patients. Squamous cell carcinoma, which has a different biological behavior, was excluded [30]. Studies were excluded from our analysis if they did not meet the above inclusion criteria, or the study population included diseases other than GC (eg, esophageal carcinoma, adenocarcinoma of esophagogastric junction [type I]) unless the data were presented separately, or it was impossible to extract or calculate appropriate data from the published results.

### Types of Interventions

Any method of chemotherapy initially performed before gastrectomy, with or without further postoperative chemotherapy (if there existed, then the postsurgical management, including regimen, and administration route, dose and schedule, had to be comparable between two groups) was included and referred as the NAC group, regardless of specific regimen, dosage and administration. As SA we considered all procedures as “surgery alone” or “primary surgery” and performed merely through gastrectomy. Processes in which further adjuvant postsurgical chemotherapy comparable between two groups were used to guarantee the efficacy were not excluded. Studies that included other types of malignancies or operation (eg, laparoscopic gastrectomy), or those that contained multivisceral resections were excluded unless the data were presented separatively.

### Outcomes of Interest and Definitions

Primary outcomes were 3-year and 5-year survival rates, overall survival time, perioperative mortality, and deaths due to recurrence/progression at the end of follow up. Secondary outcomes included down-staging effects namely tumor (ypT0-2) and nodal stages (ypN0) upon resection, R0 resection rate, and postoperative complications. Safety and toxity analysis focused on adverse effects of NAC was also conducted. As described in the included trials, survival time was recorded as the time from the date of randomization to death. Tumor and nodal stages at resection were recorded according to the 14^th^ edition of the Union for International Cancer Control (UICC) tumor node metastatic (TNM) classification of malignant tumors and the Japanese Gastric Cancer Classification [10], [28]. Objective response to NAC was evaluated as complete response (CR), partial response (PR), stable disease (SD) and progressive disease (PD) according to the criteria of Japanese Gastric Cancer Association [28].

### Data Extraction

Titles and abstracts of all retrieved records, and subsequently full-text articles were examined independently by 2 authors (A.M.X. and L.H.) according to PRISMA [26], [27] guideline. The following data were extracted separately by the same 2 authors for all included studies: reference of study, study population characteristics, study design, and inclusion and exclusion criteria. For dichotomous outcomes, the number of events was recorded and for continuous outcomes, means and standard deviations (SDs) were registered. Population characteristics include number of participating subjects, regimen of NAC performed, age and gender. In case of discrepancies, a third author was consulted and agreement was reached by consensus.

Missing data were handled by the following methods. Missing SDs were imputed on the basis of ranges when available [31]. If both means and SDs were missing, they were imputed on the basis of the medians and ranges or on the basis of medians and interquartile ranges, according to availability [31]. If neither a range nor any other measure of dispersion was available, then the SD was estimated by halving the mean or the median.

### Risk of Bias Assessment

Risk of bias was assessed for all articles by individual components using both The Cochrane Collaboration's tool for assessing risk of bias and the Jadad scoring system [32], [33]. High-quality trials scored more than 2 out of a maximum possible score of 5, while low-quality trials scored 2 or less. These assignments were made before the start of the study.

### Statistical Analysis

This study was carried out in line with the recommendations of the PRISMA [26], [27] statement. Statistical analyses were performed following the recommendations of The Cochrane Collaboration Guidelines [34]. Outcomes reported by two or more studies were pooled in meta-analyses. Our study was based on intention to treat analysis.

Dichotomous and continuous outcomes were presented as risk ratios (RRs) and weighted mean differences (WMDs), respectively. Data were pooled using the Mantel-Haenszel and the inverse-variance methods for dichotomous and continuous outcomes, respectively. Trials with zero events in both arms were excluded from meta-analysis. For all analyses, the 95% confidence interval (CI) was calculated. Heterogeneity was calculated using Higgins *χ^2^* test [35], and inconsistency in study effects was quantified by *I^2^* values [36]. The fixed-effects model was used if no heterogeneity was present (*χ^2^P*>0.100 and *I^2^*<50%). If excessive heterogeneity was present, data were first rechecked and the DerSimonian random-effects model was used when heterogeneity persisted [37]. Funnel plots were used to help identify the presence of publication or other types of bias [38], [39]. For pre-specified patient subgroup analyses stratified for pretreatment TNM stage, we additionally investigated treatment by subgroup interaction term following Fisher [40]. Review Manager software (RevMan© v. 5.0) provided by The Cochrane Collaboration was used for data management and statistical analyses.

## Results

### Selected RCTs Characteristics

A total of 347 potential relevant publications were identified (Fig. 1). We then identified 36 full-text articles comparing NAC with SA and found 17 studies did not randomly allocate patients, 7 with imbalanced postsurgical handling and 3 with inseparable data. Ychou's multicenter phase III trial [8] which assessed the comprehensive effects of preoperative combined with postoperative chemotherapy compared with SA among patients suffering from resectable adenocarcinoma of the stomach or lower esophagus was excluded for not strictly matching our qualified standards. Finally, nine original RCTs [41]–[49] comparing NAC with SA when treating GC which met the eligibility criteria were identified. Zhao's trial [47] had 3 arms comparing 2 different preoperative chemotherapy regimens with 1 control group, and for this trial (n = 60) we combined the treatment arms into 1 (n = 40) and compared this arm with the control group (n = 20); Imano's study [48] included 4 arms comparing 3 different NAC regimens with 1 control group, and for this trial (n = 63) we also combined the treatment arms into 1 (n = 47) and compared this arm with the control group (n = 16).

The 9 included RCTs were published between 1995 and 2010, with 36 to 83 months of follow-up. A total of 1056 patients were included in our analysis with 511 recieving NAC (48.4%) and 545 (51.6%) undergoing SA. Patients' characteristics are listed in Table 1 and 2. All patients had proof of GC on pathology and/or symptoms and/or signs and/or preoperative imaging and/or laboratory studies (Table 3). Matching of demographic factors was almost complete and all studies were adequately matched in the factors reviewed (Table 1). Before gastrectomy, NAC and SA groups did not differ significantly in terms of age (60.63 vs 63.37, *Z* = 1.55, *P* = 0.12) or gender (male percentage, 68.78% vs 71.17%, *Z* = 0.31, *P* = 0.76).

**Table 1:** Details of Included RCTs Comparing NAC with SA in Our Meta-Analysis (Part A).

Authors/Trial acronym	Year, Ethnicity	Accrual period	Countries where conducted	Intention to treat analysis	Matched Factors^†^	Sample Size
Shchepotin et al [41]	1995, Ukraine	NR	Ukraine (single-center)	NR	1, 2, 5, 6	97
Kang et al [42]	1996, Korea	NR	Korea (single-center)	NR	1, 2, 3, 4, 5, 7	107
Kobayashi et al [43]	2000, Japan	1990–1993	Japan (multi-center)	No	1, 2, 11	171
Wang et al [44]	2000, China	1987–1988	China (single-center)	NR	1, 2, 3, 4, 11	60
Hartgrink et al/FAMTX [45]	2004, Holland	1993.9–1996.1	Netherlands (multi-center)	No	1, 4, 5, 6, 10	59
Nio et al [46]	2004, Japan	1991–1999	Japan (single-center)	No	1, 2, 3, 4, 5	295
Zhao et al [47]	2006, China	2001.10–2005.3	China (bi-center)	No	1, 2, 5, 6, 7, 8	60
Imano et al [48]	2010, Japan	1992–2002	Japan (single-center)	Yes	1, 2, 4, 5, 9, 10	63
Schuhmacher et al/EORTC 40954 [49]	2010, Germany	1999.7–2004.2	Several European countries and Egypt (multi-center)	Yes	1, 2, 4, 5, 9, 10, 11, 12, 13	144

RCTs, randomized controlled trials; NAC, neoadjuvant chemotherapy; SA, surgery alone; NR, not reported; EORTC, European Oraganisation for Research and Treatment of Cancer.

† Matching: 1, age; 2, gender; 3, histological grade; 4, lymphonectomy; 5, way of gastrectomy; 6, leukocyte count; 7, haematoglobin; 8, thromboplastin; 9, tumor location; 10, histological type; 11, T stage; 12, N stage; 13, M stage.

**Table 2:** Details of Included RCTs Comparing NAC with SA in Our Meta-Analysis (Part B).

Authors	Main inclusion criteria	Regimen and administration	Median Follow-up (months)	Available outcomes
Shchepotin et al [41]	Gastric carcinoma	Intra-arterial	NR	OS
Kang et al [42]	Gastric adenocarcinoma	PEF (DDP/epirubicin/5-FU)	>36	R0 resection, tumor stage at resection
Kobayashi et al [43]	Resectable advanced gastric cancer; ≤75 years	5′-DFUR: oral, ≥610 mg/m^2^/d×10 d	NR	OS, R0 resection, tumor stage at resection (only T stage), safety of NAC
Wang et al [44]	Resectable gastric cancer	FPLC: oral	60	5-year survival, perioperative morbidity
Hartgrink et al [45]	Resectable gastric adenocarcinoma; >cT1 M0; PS 0–2; ≤75 years	FAMTX: intravenous; methotrexate 1500 mg/m^2^, 5-FU 1500 mg/m^2^, leucovorin 30 mg/6 h×2 d, doxorubicin 30 mg/m^2^; 4 courses	83	OS, R0 resection, tumor stage at resection, safety of NAC, perioperative morbidity
Nio et al [46]	Resectable gastric cancer; PS 0–3	UFT (tegafur/uracil): oral, FT: 7 mg/kg/d×21 d	83	OS, R0 resection, tumor stage at resection, safety of NAC, perioperative morbidity
Zhao et al [47]	Gastric adenocarcinoma, Karnofsky's scale >90, ≤70 years	5′-DFUR (oral, 800–1200 mg/d) or DDP/5-FU (intravenous, 500 mg 5-FU+200 mg/d CF)×3–5 d	NR	OS, R0 resection, tumor stage at resection, perioperative morbidity and mortality
Imano et al [48]	Resectable advanced gastric cancer; <75 years; PS 0–1	5-FU (330 mg/m^2^/d×3 d) or DDP (18 mg/m^2^) or 5-FU+DDP: intravenous	NR	OS, R0 resection, tumor stage at resection, safety of NAC, perioperative morbidity
Schuhmacher et al [49]	Locally advanced resectable gastric adenocarcinoma, stages III and IV; cT3/4 M0/1; PS 0–1; 18–70 years	DDP (50 mg/m^2^/d×3 d), d-L-folinic acid (500 mg/m^2^/d×6 d), 5-FU (2000 mg/m^2^/d×6 d); 2 courses; intravenous	53	OS, progression-free survival (PFS), R0 resection, tumor stage at resection, safety of NAC, perioperative morbidity

RCTs, randomized controlled trials; NAC, neoadjuvant chemotherapy; SA, surgery alone; NR, not reported; OS, overall survival; PS, performance status (ECOG/WHO); 5-FU, 5-fluorouracil; 5′-DFUR, 5′-Deoxy-5-fluorouridine; DDP, cisplatin; FPLC, fluorouracil polyphase liposome composita pro orale, consisting of 5-FU, oleic acid, ginseng polysaccharides, bean phospholipid and cholesterol; R0 resection, presence of tumor-free resection margin.

**Table 3:** Criteria for Gastric Cancer Inclusion Eligibility and Assessment.

Authors	Symptoms and signs	Endoscopy/Pathology	Preoperative imagings	Laboratory studies	Severe comorbidities	Previous therapy	Other malignacies
Shchepotin et al [41]	Yes	Yes	Yes	Yes	No	No	NR
Kang et al [42]	NR	Yes	NR	Yes	NR	NR	NR
Kobayashi et al [43]	Yes	Yes	No	Yes	No	No	NR
Wang et al [44]	Yes	Yes	Yes	NR	No	NR	NR
Hartgrink et al [45]	Yes	Yes	Yes	Yes	No	NR	No
Nio et al [46]	Yes	Yes	Yes	Yes	No	No	No
Zhao et al [47]	Yes	NR	NR	NR	NR	NR	NR
Imano et al [48]	NR	Yes	Yes	Yes	No	NR	No
Schuhmacher et al [49]	NR	Yes	Yes	NR	No	No	No

NR, not reported.

### Methodological Quality Assessment

The trials had fair methodological quality with a mean Jadad score of 2.33 (range, 1–4). They mostly suffer from methodological drawbacks frequently seen in clinical RCTs in general, mainly difficulties in concealing the allocation of patients, the inherent complexity of blinding between two procedures and small number of patients included in part of the researches. All trials had adequate sequence generation. Seven trials did not report double allocation concealment and 2 did not report loss to follow-up. Seven trials reported a sample size calculation (Table 4). Particular features including primary endpoint, original clinical stage, percentages of D2 resections and of patients subjecting to a curative operation which may have an additional impact on the quality of the analyzed trials were shown in table 5.

**Table 4:** Quality Assessment and Risk of Bias Summary.

	Shchepotin et al [41]	Kang et al [42]	Kobayashi et al [43]	Wang et al [44]	Hartgrink et al [45]	Nio et al [46]	Zhao et al [47]	Imano et al [48]	Schuhmacher et al [49]
Adequate sequence generation?	Yes	Yes	Yes	Yes	Yes	Yes	Yes	Yes	Yes
Allocation concealment?	Unclear	Unclear	Yes	Unclear	Yes	No	Unclear	Unclear	Unclear
Blinding (observer)?	Unclear	No	Yes	No	Unclear	No	Unclear	Unclear	Unclear
Blinding (patient)?	Unclear	No	Unclear	No	Unclear	No	Unclear	Unclear	Yes
Incomplete outcome data addressed?	No	Yes	Yes	Yes	No	Unclear	No	No	No
Postoperative protocol reported?	Yes	Yes	Yes	Yes	Yes	Yes	Yes	Yes	Yes
Adequate report on loss to follow-up?	Yes	Yes	Yes	Yes	Yes	Yes	Unclear	Unclear	Yes
Free of selective reporting?	Yes	Yes	Yes	Yes	Yes	Yes	Yes	Yes	Yes
Free of other bias?	Yes	Yes	Yes	Yes	Yes	Yes	Yes	Yes	Yes
Sample size calculation?	No	Yes	Yes	Yes	Yes	Yes	Yes	No	Yes
Jadad score	2	2	4	2	4	1	1	2	3

**Table 5:** Features with Possible Additional Impact on Analyzed Trial Quality.

Authors	Primary endpoint	Method	Original clinical stage	Percentage of D2 resections	Percentage of patients subjecting to curative operation
Shchepotin et al [41]	Overall survival	NAC	NR	NR	62%
		SA	NR	NR	NR
Kang et al [42]	NR	NAC	NR	NR	70%
		SA	NR	NR	61%
Kobayashi et al [43]	Overall survival	NAC	NR	NR	46%
		SA	NR	NR	59%
Wang et al [44]	Overall survival	NAC	NR	NR	NR
		SA	NR	NR	NR
Hartgrink et al [45]	Curative resectability	NAC	NR	0	67%
		SA	NR	0	66%
Nio et al [46]	Overall survival	NAC	I, 46.1%; II, 14.7%; III, 14.7%; IV, 24.5%	55.9%	NR
		SA	I, 63.7%; II, 9.3%; III, 13.0%; IV, 14.0%	48.2%	NR
Zhao et al [47]	NR	NAC	NR	NR	70% (overall)
		SA	NR	NR	
Imano et al [48]	NR	NAC	NR	100%	NR
		SA	NR	100%	NR
Schuhmacher et al [49]	Overall survival	NAC	T3, 86.1%; T4, 11.1%; N0, 5.6%; N1, 66.7%; N2, 8.3%; N3, 1.4%; M0, 91.7%; M1, 1.4%	95.7%	87.5%
		SA	T3, 88.9%; T4, 9.7%; N0, 8.3%; N1, 61.1%; N2, 6.9%; N3, 1.4%; M0, 95.8%; M1, 1.4%	92.6%	87.5%

NAC, neoadjuvant chemotherapy; SA, surgery alone; NR, not reported.

### Primary Outcomes

Detailed data and analyses by categories are available in Table 6 and 7.

**Table 6:** Primary Outcomes.

Authors	Method	n	3-year survival	5-year survival	Survival months	Total mortality	Perioperative mortality	Death due to recurrence/progression
Shchepotin et al [41]	NAC	47	42	37	NR	10	NR	NR
	SA	50	18	15	NR	35	NR	NR
Kang et al [42]	NAC	53	NR	NR	NR	NR	NR	NR
	SA	54	NR	NR	NR	NR	NR	NR
Kobayashi et al [43]	NAC	91	NR	58	NR	33	NR	NR
	SA	80	NR	52	NR	28	NR	NR
Wang et al [44]	NAC	30	NR	12	NR	18	0	NR
	SA	30	NR	7	NR	23	0	NR
Hartgrink et al [45]	NAC	29	9	6	18.2	24	2	12
	SA	30	14	10	30.3	20	1	8
Nio et al [46]	NAC	102	78	73	NR	29	NR	NR
	SA	193	143	137	NR	66	NR	NR
Zhao et al [47]	NAC	40	19	NR	NR	13	0	NR
	SA	20	11	NR	NR	9	0	NR
Imano et al [48]	NAC	47	26	20	NR	27	0	NR
	SA	16	8	6	NR	10	0	NR
Schuhmacher et al [49]	NAC	72	47	39	64.62	32	3	24
	SA	72	36	34	52.53	35	1	33

NAC, neoadjuvant chemotherapy; SA, surgery alone; NR, not reported.

**Table 7:** Analysis of Primary and Secondary Outcomes by Categories.

Category	No. RCTs	NAC	SA	RR	WMD	95% CI	*P*
3-year survival	6	221/337 (65.58%)	230/381 (60.37%)	1.18		0.86–1.61	0.30
5-year survival	7	245/418 (58.61%)	261/471 (55.41%)	1.20		0.93–1.56	0.17
Survival months	2	51.29 (n = 101)	45.99 (n = 102)		−0.29	−23.98 to 23.41	0.98
Total mortality	8	186/458 (40.61%)	226/491 (46.03%)	0.83		0.65–1.06	0.14
Perioperative mortality	5	5/218 (2.29%)	2/168 (1.19%)	2.54		0.50–12.77	0.26
Death due to recurrence/progression	2	36/101 (35.64%)	41/102 (40.20%)	0.89		0.62–1.26	0.50
Tumor stage upon resection (ypT0-2)	6	236/394 (59.90%)	183/445 (41.12%)	1.24		0.80–1.92	0.34
Nodal stage upon resection (ypN0)	3	38/148 (25.68%)	20/118 (16.95%)	1.92		1.20–3.06	0.006
R0 resection	4	154/245 (62.86%)	147/236 (62.29%)	1.02		0.89–1.17	0.81
Postoperative complications	6	41/320 (12.81%)	48/391 (12.28%)	1.14		0.77–1.70	0.51

RCTs, randomized controlled trials; NAC, neoadjuvant chemotherapy; SA, surgery alone; RR, risk ratio; WMD, weighted mean difference; 95% CI, 95% confidence interval.

### Survival

**Figure 2:**
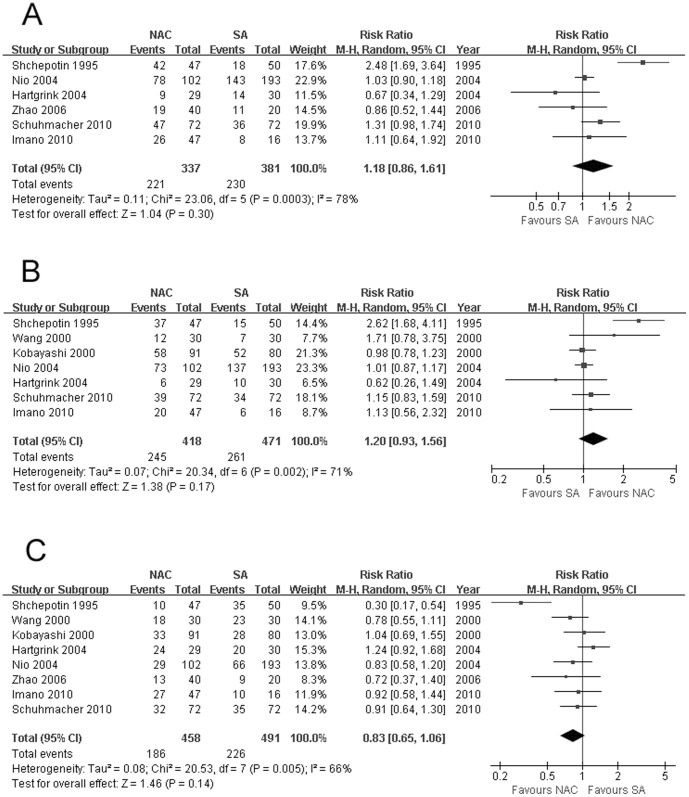
(A) 3-year survival, (B) 5-year survival, and (C) overall mortality by NAC and SA procedures, all showing no significant difference. The relative weight of each study is proportional to the size of the corresponding box in the Forest plot. NAC, neoadjuvant chemotherapy; SA, surgery alone.

Results for 3 and 5 years were available for 6 and 7 RCTs respectively. Both studies had significant heterogeneity (*χ^2^* = 23.06, *P* = 0.0003, *I^2^* = 78%; *χ^2^* = 20.34, *P* = 0.002, *I^2^* = 71%) between two groups, so random-effects model was chosen. No significant difference was observed for both parameters between NAC and SA when treating GC (65.58% vs 60.37%, RR: 1.18, 95% CI: 0.86–1.61, *P* = 0.30, Fig. 2A; 58.61% vs 55.41%, RR: 1.20, 95% CI: 0.93–1.56, *P* = 0.17, Fig. 2B). Nio [46] showed that the survival benefit for NAC was only significant in stage 2 or 3 patients with response to chemotherapy. However, Hartgrink [45], member of Dutch Gastric Cancer Group, revealed that for patients operated with curative intent, SA group showed obvious larger survival benefits than NAC group (5-year survival rate: 53% vs 32%, median survival months: 66 vs 30). However, pooled analysis of the 2 reports [45], [46] comparing two methods dealing with GC in diverse stages separately revealed that 5-year survival rates were all comparable (Stage I: 89.29% vs 90.63%, RR: 1.04, 95% CI: 0.95–1.13, *P* = 0.45; Stage II: 57.14% vs 60.71%, RR: 0.91, 95% CI: 0.56–1.50, *P* = 0.72; Stage III: 57.89% vs 36.67%, RR: 1.62, 95% CI: 0.90–2.92, *P* = 0.11; Stage IV: 21.21% vs 11.11%, RR: 1.89, 95% CI: 0.63–5.69, *P* = 0.26) without significant heterogeneity, with a small number of patients analyzed though. Kobayashi [43] further reported that the 5-year survival rate of patients showing good compliance with NAC was significantly higher than that of patients with poor compliance (53.3% vs 22.0%). Survival months provided by 2 trials [45], [49] also revealed no significant difference between 2 procedures (51.29 vs 45.99, WMD: −0.29, 95% CI: −23.98 to 23.41, *P* = 0.98) with random-effects model applied due to significant heterogeneity (*χ^2^* = 16.92, *P*<0.0001, *I^2^* = 94%). Shchepotin [41] found that pre-operative intravenous (systematic) chemotherapy (IVCH) produced no survival benefit compared with SA without detailed data provided. Shuhmacher's progression-free survival analysis [49] based on 44 events observed in the NAC arm versus 40 in the SA arm revealed no significant difference, and the HR comparing NAC versus SA was 0.76 (95% CI, 0.49 to 1.16; *P* = 0.20).

### Mortality

There being significant heterogeneity (*χ^2^* = 20.53, *P* = 0.005, *I^2^* = 66%), random-effects model chosen showed that there tended to be higher rate of mortality among patients undergoing SA than those receiving NAC at the end of follow-up (8 RCTs, 46.03% vs 40.61%, RR: 0.83, 95% CI: 0.65–1.06, *P* = 0.14, Fig. 2C). Perioperative mortality and death due to recurrence/progression were further analyzed, both revealed similar results between NAC and SA with fixed-effects model used thanks to insignificant heterogeneity (8 RCTs, 2.29% vs 1.19%, RR: 2.54, 95% CI: 0.50–12.77, *P* = 0.26; 2 RCTs, 35.64% vs 40.20%, RR: 0.89, 95% CI: 0.62–1.26, *P* = 0.50).

### Secondary Outcomes

Detailed data and analyses by categories are available in Table 7 and 8.

**Table 8:** Secondary Outcomes.

Authors	Method	n	Tumor stage upon resection (ypT0/1/2)	Nodal stage upon resection (ypN0)	R0 resection	Postoperative complications
Shchepotin et al [41]	NAC	47	NR	NR	29	NR
	SA	50	NR	NR	NR	NR
Kang et al [42]	NAC	53	14	NR	37	NR
	SA	54	9	NR	33	NR
Kobayashi et al [43]	NAC	91	57	NR	42	NR
	SA	80	52	NR	47	NR
Wang et al [44]	NAC	30	NR	NR	NR	0
	SA	30	NR	NR	NR	0
Hartgrink et al [45]	NAC	29	14	11	16	2
	SA	30	15	7	19	5
Nio et al [46]	NAC	102	83	NR	NR	15
	SA	193	62	NR	NR	30
Zhao et al [47]	NAC	40	NR	NR	NR	3
	SA	20	NR	NR	NR	1
Imano et al [48]	NAC	47	22	0	NR	2
	SA	16	11	0	NR	1
Schuhmacher et al [49]	NAC	72	46	27	59	19
	SA	72	34	13	48	11

NAC, neoadjuvant chemotherapy; SA, surgery alone; R0 resection, resection with tumor-free margin; NR, not reported.

### Down-staging Effect

**Figure 3:**
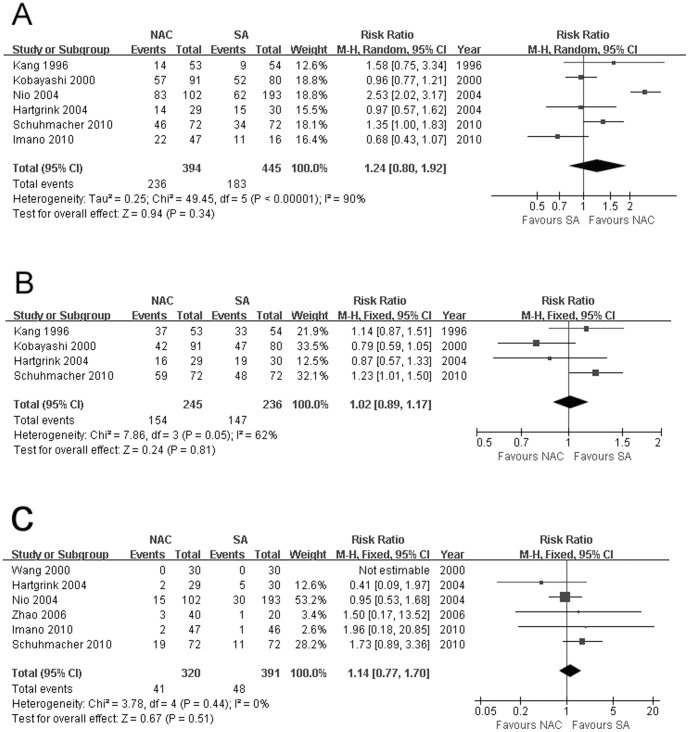
(A) Tumor stage upon resection (ypT0-2), (B) presence of tumor-free resection margin, and (C) postoperative complications, all showing comparable results between NAC and SA processes. The relative weight of each study is proportional to the size of the corresponding box in the Forest plot. NAC, neoadjuvant chemotherapy; SA, surgery alone.

Since there was significant heterogeneity (*χ^2^* = 49.45, *P*<0.00001, *I^2^* = 90%) for tumor stage upon resection (ypT0-2), random-effects model was applied. The combined data from 6 trials demonstrated comparable results between two groups (59.90% vs 41.12%, RR: 1.24, 95% CI: 0.80–1.92, *P* = 0.34, Fig. 3A). Shchepotin [41] found an impressive rate of 61.6% with no residual tumor in the resected stomach, while the data in the SA group was not accessible. No significant heterogeneity observed for nodal stage upon resection (ypN0), fixed-effects model was used, and pooled result revealed that there was significantly more ypN0 status achieved among patients treated with NAC than SA (25.68% vs 16.95%, RR: 1.92, 95% CI: 1.20–3.06, *P* = 0.006).

### Presence of Tumor-free Resection Margin

**Figure 4:**
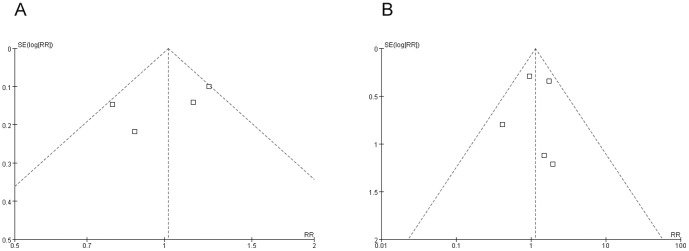
Funnel plots for (A) presence of tumor-free resection margin, and (B) postoperative complications, showing that both are free from publication bias. RR, relative risk; SE, standard error.

There existing no significant herterogeneity or bias demonstrated by funnel plot (Fig. 4A), analysis with a fixed-effects model sustained that NAC didn't hopefully result in a significantly higher incidence of R0 resection compared with SA (4 RCTs, 62.86% vs 62.29%, RR: 1.02, 95% CI: 0.89–1.17, *P* = 0.81, Fig. 3B).

### Postsurgical Complications

Funnel plot supporting no bias (Fig. 4B) and heterogeneity not existing, fixed-effects model showed that postoperative morbidities between two procedures were similar (6 RCTs, 12.81% vs 12.28%, RR: 1.14, 95% CI: 0.77–1.70, *P* = 0.51, Fig. 3C).

### Analysis of Adequate Quality Trials with Combination Chemotherapy Regimens

Hartgrink's [45] and Schuhmacher's trials [49] were separately analyzed. There being no significant heterogeneity, fixed-effects model chosen revealed no significant difference between two groups in 3-year (55.45% vs 49.02%, RR: 1.13, 95% CI: 0.87–1.47, *P* = 0.37) or 5-year survival rate (44.55% vs 43.14%, RR: 1.03, 95% CI: 0.76–1.40, *P* = 0.85), total (55.45% vs 53.92%, RR: 1.03, 95% CI: 0.81–1.32, *P* = 0.80) or perioperative mortality (4.95% vs 1.96%, RR: 2.54, 95% CI: 0.50–12.77, *P* = 0.26), or postsurgical complication (20.79% vs 15.69%, RR: 1.32, 95% CI: 0.73–2.39, *P* = 0.36). NAC tended to result in more ypT0-2 status upon resection (59.41% vs 48.04%, RR: 1.24, 95% CI: 0.95–1.60, *P* = 0.11) and R0 resection (74.26% vs 65.69%, RR: 1.13, 95% CI: 0.94–1.35, *P* = 0.19), and significantly contributed to higher incidence of ypN0 upon resection (25.68% vs 16.95%, RR: 1.92, 95% CI: 1.20–3.06, *P* = 0.006) when compared to SA.

### Others

Kobayashi [43] found that pre-operative oral administration of 5′-Deoxy-5-fluorouridine (5′-DFUR) reduces hematogeneous metastasis of GC. Wang [44] revealed that pre-surgical FPLC treatment could reduce the number of tumor emboli while increase cell degeneration and necrosis, thus inhibiting tumor proliferative, invasive and metastatic activities, and stimulating the patient's immune system. Zhao's study [47] showed that preoperative oral 5′-DFUR administration may induce apoptosis of gastric carcinoma cells, and decrease tumor cell proliferation index. Imano [48] also found that combination of cisplatin (DDP) and 5-FU reduced proliferative potency and increased cellular apoptosis in gastric cancer cells. Pooled analysis was not available on these parameters.

### Objective Response to NAC

The overall NAC response rate (CR+PR) was calculated to be 43.0% (105/244), as was show in Table 9. Hartgrink [45] found that low response seemed to lead to a decreased prognosis.

**Table 9:** Objective Response to Neoadjuvant Chemotherapy.

Authors	n	CR+PR	SD+PD
Shchepotin et al [41]	47	41 (87.1%)	6 (12.9%)
Hartgrink et al [45]	25	8 (32%)	17 (68%); 10 SD+7 PD
Nio et al [46]	87	29 (33.3%); 2 CR+27 PR	58 (66.7%); 58 CR+0 PD
Imano et al [48]	16	1 (6.25%); 0 CR+1 PR	15 (93.75%); 15 SD+0 PD
Schuhmacher et al [49]	69	26 (37.68%); 5 CR+21 PR	43 (62.32%); 39 SD+4 PD

CR, complete response; PR, partial response; SD, stable disease; PD, progressive disease.

### Safety Analysis

Safety analysis included both NAC-induced adverse effects (defined according to the Common Toxicity Criteria of the National Cancer Institute). Hartgrink [45] reported toxicity happened to 5 (17.2%) patients during NAC. According to Nio [46], a total of 24 (23.5%) patients experienced NAC-related grade3/4 side-effects, including anorexia, leukopenia, thrombocytopenia, liver dysfunction and massive bleeding from GC. In Imano's study [48], no severe side effects of NAC happened. Schuhmacher [49] reported 8 (32%) patients experiencing toxicity (2 renal toxicity [maximum grade 2], 1 cardiac toxicity [grade 3], 4 nausea [maximum grade 3] and vomiting [maximum grade 3], and 1 neutropenia [grade 2]). The comprehensive rate was 18.2% (37/203).

### Sensitivity Tests

**Figure 5:**
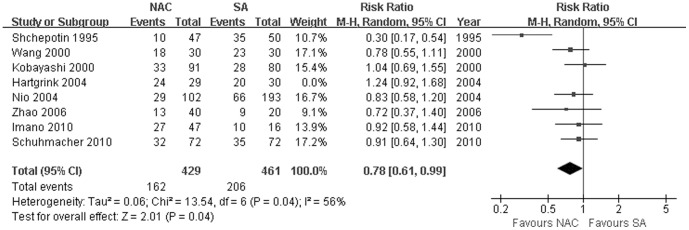
Sensitivity test for total mortality between NAC and SA measurements, showing that there existed significantly lower mortality rate among patients receiving NAC than those undergoing SA when Hartgrink's study was excluded. The relative weight of each study is proportional to the size of the corresponding box in the Forest plot. NAC, neoadjuvant chemotherapy; SA, surgery alone.

There were significantly lower total mortality rates for patients receiving NAC than those undergoing SA when Hartgrink's study [45] was excluded (37.76% vs 44.69%, RR: 0.78, 95% CI: 0.61–0.99, *P* = 0.04, Fig. 5). Sensitivity analyses of all the other outcomes yielded similar results. Funnel plots and an exhaustive and strict literature search conferred a substantial degree of confidence in our pooled findings.

## Discussion

Chemotherapy is an adjuvant treatment modality in the form of adjuvant chemotherapy, NAC and concomitant chemoradiotherapy [50]. For GC patients, adjuvant chemoradiotherapy is the standard treatment in the US, perioperative chemotherapy is the first choice in Europe, surgery combined with adjuvant chemotherapy is recommended in Japan where D_2_ gastrectomy is effective and safe, and D2 plus postoperative adjuvant chemotherapy with capecitabine and oxaliplatin for around 6 months in Korea [6], [11], [51]. Although a number of phase III studies have been reported in the last few decades, the best regimen of postoperative chemotherapy remains a point of argue and active research [13]. Adjuvant chemotherapy using S-1 for 12 months has recently been established as the standard treatment after D2 gastrectomy in Japanese patients with Stage II or III disease based on a large phase III study [10]. Kodera [52] reported that a 2-year survival rate of 46% was obtained with surgery and S-1 therapy in patients with CY1. The standard regimen administered for metastatic disease is combination chemotherapy using S-1 plus DDP (SC) which was established from a Phase III trial [10], [28]. Recently, the feasibility of SC was tested in an adjuvant setting to see whether this combination regimen is suitable for a test arm of a future Phase III trial which revealed that SC was not tolerable when it was started just after surgery, but was feasible and safe when provided preoperatively [53]–[57]. Paclitaxel is another key drug used for metastatic disease and has been tested in an adjuvant setting in a phase III trial [58], [59]. Moreover, paclitaxel plus DDP (PC) demonstrated a high response rate and feasibility for metastatic disease [58]. Furthermore, PC achieved a high pathological response rate with acceptable toxicity in the neoadjuvant setting [60]. Two courses have been selected in most Japanese studies, while three courses were adopted in the MAGIC phase III trial, which confirmed its survival benefit [15], [55]. The optimal approach in individual patients remains controversial [19].

NAC has several advantages including good toleration, better control of micrometastasis, and potentiality to downstage tumor and increase the probability of R0 resection so as to facilitate surgery [61]. NAC has been proven effective against some cancers, especially breast cancer [62]–[65]. Since Wilke [14] initially conducted NAC when treating GC in1989, there've been many trials evaluating this new method mainly among resectable advanced GC patients without metastasis and many reported ideal achievements. However, most of reports are limited to nonrandomized retrospective study based on relatively small population and focus on the aspect of regimen.

The results of RCTs on NAC versus SA for GC vary in aspects of efficacy and safety. Four systematic reviews with considerable defects also made discrepant conclusions [17]–[20]. Compared with the previous studies, our analyses share some similarities. But the recall and precision ratios of literature search have a great impact on the accuracy of pooled estimates, and the previous meta-analyses comparing NAC with SA contain ineligible studies, thus leading to great bias. There also existed major limitations in the included RCTs in this analysis, including the absence of endoscopic ultrasonography in preoperative clinical staging, the heterogeneity in some outcomes, the single agent or inferior combination regimen used in most trials, and the diverse, maybe less effective schedule of chemotherapy.

This study summarizes the highest quality data comparing NAC with SA. In our analysis, RCTs were all published after 1995, and those published after 2000 constitute most of the studies included. Some of the individual trials were inconclusive as they were underpowered and hence too small to identify the important determinants of ideal NAC. This meta-analysis aims to provide this evidence. The methodological quality of the 9 RCTs included in this meta-analysis was fair. Regimens of the included trials were standardized. Study population was similar between trials in all mentioned aspects.

Ge's [18] and Li's studies [19] demonstrated minor but significant benefits in patient survival, and a phase II trials [15] clarified that a high 3-year survival rate was obtained with NAC: 27% with two courses of CPT-11 plus DDP. However, Liao's [17] and Wu's analyses [20] and a recent trial [66] demonstrated that NAC and D2 surgery could not effectively improve the overall survival. The convincing level 1a evidence provided by us showed that no significant differences existed in 3-year or 5-year survival, post-management living period, total death, or mortality due to recurrence/progressive disease, which may be because NAC, although inhibiting malignancy proliferation and promoting lesion necrosis, leads to weakening of immune system and delay of prompt curative management. Although not significant, stage III and IV GC patients tended to have better 5-year survival rates with NAC than SA, while in stages I and II, the rates seemed slightly worse with NAC. This may indicate that NAC could be beneficial in advanced stages, which needs to be addressed by longer follow-up period and larger sample size. Perioperative mortality rates of this study were 2.29% for NAC and 1.19% for SA which were comparable. Intervals between randomization/NAC and surgery and regimens may be potential influential factors impacting parameters of efficacy and safety though. Previously, several investigators reported that the pathological response clearly separated the survival of GC patients who received NAC [67]. A better outcome than expected after radical SA due to the widespread high quality of surgery with resections of regional lymph nodes outside the perigastric area (D2) may also conceal part of effects. Patients subjecting to curative resection took up the majority part in selected trials. However, percentage of D2 resection varied greatly in different periods and countries. When we excluded Hartgrink's results [45], we found that NAC contributed to significantly lower overall mortality, which might be due to the relatively inferior combination applied [68], the relatively long interval between randomization and operation in the NAC group, curative respectability being the primary endpoint, and the fact that all patients underwent D1 gastrectomy in the study. In GC patients, combination therapy is related with a significant survival benefit compared to single agent therapy [69]. However, single agent or inferior combination therapy was applied in most of the RCTs available [68], which might impact our results greatly. Administration of the most effective chemotherapeutic regimens is essential in the case of a neoadjuvant manipulation. Furthermore, several European Phase III trials have demonstrated that 2 or 3 courses of NAC, followed by curative surgery and 3 or 4 courses of adjuvant chemotherapy using 5-FU plus DDP, significantly improved overall and disease-free survival for patients with resectable adenocarcinoma of the stomach compared with surgery alone [8], which requires novel pooled analysis to make conclusion more persuasive on perioperative chemotherapy. Importantly, a significant percentage of patients (34.4%) in the perioperative chemotherapy arm of the MAGIC trial [23] did not receive the “adjuvant” part, and much of the positive outcome might be attributed to the effect of the neoadjuvant manipulation.

NAC, which is brought about to improve resection condition, is under heated discussion about its definite role in improving cure rate for GC patients [17], [18]. European Organization for Research and Treatment of Cancer Randomized Trial 40954 [49] showed a significantly increased R0 resection rate. According to our convincing analysis, stronger nodal down-staging effect was observed with NAC performed, which is the main difference between NAC and SA, while other benefits for resectability weren't firmly demonstrated. This is in contrast with findings of systematic reviews reporting that NAC and SA share all same clinical outcomes [17], [20]. There were basically no significant differences in outcome measures of R0 resection and postoperative morbidities. Still, it's notable that accuracy of staging laparoscopy was 71.4% for T staging and 75.9% for N staging [70]. Moreover, the MAGIC trial [23] reported that the perioperative-chemotherapy group was accompanied with significantly smaller tumor maximum diameter, a greater proportion of stage T1 and T2 tumors, and a significant trend to less advanced nodal disease (N0/1) compared to the surgery group. A French trial reported that perioperative therapy increased the curative resection rate [8].

Lack of response to NAC may delay curative surgery, and chemotherapy-induced toxicity may lead to increased surgical complications [71]. Ychou [8] reported frequent grade 3/4 adverse effects of NAC, including gastrointestinal side effect and leucopenia, while Li [19] argued it was accompanied with rather good safety, and several Phase II studies have also demonstrated that SC was safe and feasible in the neoadjuvant setting [57]. Our high-quality evidences revealed that the overall NAC response rate (CR+PR) was 43.0%, and the comprehensive NAC-related adverse-effect rate was estimated to be 18.2%. The great variability of the objective response rates in included trials might be due to issues of interval between administration and gastrectomy, trial type and phase, and administration route. The trial of Imano et al [48] had the lowest rate, which could be justified from the fact that it was primarily a translational trial and chemotherapy was administered for 72 h before gastrectomy, while in Shchepotin's study [41], which had the highest rate, intra-arterial chemotherapy was performed. An S-1/DDP/paclitaxel combination regimen showed response rates of 63.5% and 59.1% in two phase II trials [72], [73]. In Japan, paclitaxel has been tested as a second-line chemotherapy for metastatic disease in several Phase II trials [58]. On the other hand, triplet regimen using docetaxel instead of paclitaxel showed much higher response rate, 87.1% and 81.3% in two Phase II studies [74], [75]. Both paclitaxel and docetaxel have several unique characteristics, including that: (i) it is not cross-resistant with 5-FU; (ii) it is active against poorly differentiated carcinoma; (iii) it has a good transition from the blood to the peritoneal cavity; and (iv) it induces a relatively low incidence of gastrointestinal toxicities [58], [76].

Number of courses administered may be another great impact factor. In ACCORD 07 [8], 25/113 patients (22.1%) received all 4 postoperative cycles as planned. On the basis of the previous studies, a randomized phase II trial is conducted at present to test the feasibility and efficacy using 2 or 4 courses of SC and PC with a 2-by-2 factorial design for macroscopically resectable locally advanced gastric cancer [77]. So far, four courses of NAC seems to contribute to a higher pathological CR rate compared with using 1, 2, or 3 courses for GC [8], [15], [55]–[57].

Recently, a number of novel trials have been registered to examine the role of NAC in treatment of advanced GC, such as S-1 plus DDP or S-1 and DDP plus Taxanes [55], [78]. The incorporation of Taxanes into the 5-FU/DDP (FP) regimen makes up the Taxol/5-FU/DDP (TPF) regimen, which is a promising treatment strategy for GC [79]. JACCO GC-01 Phase II trial is a study of NAC for clinically resectable T4 tumors [57]. Several regimens and courses of NAC were tested in clinical T4 or clinical stage III patients in Phase II trials [77]. Another phase III multicenter study [80] is currently being conducted in the Netherlands, which enrolled patients with resectable GC. It's hoped that they will address questions better.

Therefore, NAC should not be recommended as a regular and routine treatment for GC before obtaining abundant evidences of its certain efficacy on GC, and should be applied under the framework of clinical trials. Adequate surgery (D2 or D1 gastrectomy based on racial characteristics, tumor progression, local standard, and operator's experience) without delay may remain the appropriate management for operable GC, until further large multicenter randomized studies sustaining NAC occurs. However, with joint efforts of clinicians, enterprises and academic centers, improvements in regimen like SP or PC, and maturation and modification of courses and administration, it's reasonable to believe that conducting NAC may benefit more and more GC patients with lower NAC-related adverse effects. This treatment modality is worthy of further investigation. Besides, individuality should be focused on during comprehensive treatment of GC patients, and systematic chemotherapy would be necessary among patients with micrometastatic disease already at diagnosis.

The internal validity of this study is fair, mainly affected by the quality of RCTs available, with low risk of bias though. This analysis is limited by the diverse regimens, intervals between randomization and surgery, and follow-up period and the fact that not all outcomes of interest are reported by all enrolled studies.

In conclusion, NAC doesn't contribute to significant survival benefits during the treatment of GC, and compares favorably with SA in tumor-free resection rates and postoperative complications. This may be due to regimens and courses issues. NAC definitely reduces nodal stage upon resection, and may result in a lower incidence of total mortality at the end of follow-up. All other indexes are similar. Response rate may be an important influential factor impacting possible advantages, and chemotherapy-related adverse effects can be a drawback. This level 1a evidence doesn't support NAC to outweigh SA in terms of efficacy and safety when dealing with GC. Still, further high-quality RCTs are needed to to update our finding with advancement of regimens, and future researches should be conducted in patients suffering from GC of discrepant stages and grades, and in those at diverse period of ages, separately.

## Supporting Information

Checklist S1 PRISMA checklist for this study.
(DOC)
